# Reduced Penetrance and Variable Expression of SCN5A Mutations and the Importance of Co-inherited Genetic Variants: Case Report and Review of the Literature

**DOI:** 10.1016/s0972-6292(16)30754-9

**Published:** 2014-05-25

**Authors:** T Robyns, D Nuyens, L Van Casteren, A Corveleyn, T De Ravel, H Heidbuchel, R Willems

**Affiliations:** 1 Department of Cardiovascular Medicine, University Hospitals Leuven, Leuven, Belgium; 2 Center for Human Genetics, University Hospitals Leuven, Leuven, Belgium

**Keywords:** genetic variants, Brugada syndrome, SCN5A disease

## Abstract

Mutations in the SCN5A gene are responsible for multiple phenotypical presentations including Brugada syndrome, long QT syndrome, progressive familial heart block, sick sinus syndrome, dilated cardiomyopathy, lone atrial fibrillation and multiple overlap syndromes. These different phenotypic expressions of a mutation in a single gene can be explained by variable expression and reduced penetrance. One of the possible explanations of these phenomena is the co-inheritance of genetic variants. We describe a family where the individuals exhibit a compound heterozygosity in the SCN5A gene including a mutation (R1632H) and a new variant (M858L). Individuals with both the mutation and new variant present with a more severe phenotype including spontaneous atrial tachyarrhythmia at young age. We give an overview of the different phenotypes of "SCN5A disease" and discuss the importance of co-inherited genetic variants in the expression of SCN5A disease.

## Introduction

The α-subunit of the cardiac sodium channel is called Nav1.5 and is encoded by the SCN5A gene. Mutations in this gene are known to cause different cardiac abnormalities including the channelopathies Brugada syndrome (BrS) and long QT syndrome (LQTS), but also progressive familial heart block (PFHB), sick sinus syndrome (SSS) and more recently dilated cardiomyopathy (DCM) and lone atrial fibrillation (l-AF) [1-6]. Overlap syndromes have been described involving clinical symptoms or arrhythmias of various arrhythmogenic presentations [7]. The genetic phenomena of reduced penetrance and variable expression typical of monogenic disorders also occur in the primary cardiac arrhythmia syndromes [8]. The penetrance of a disease-causing mutation is defined as the proportion of individuals with the same primary genetic defect who exhibit the associated clinical symptoms. Variable expression on the other hand is defined as the variation in clinical features (type and severity) among carriers of the same genetic defect, even within the same family.Different explanations for reduced penetrance and variable expression have been proposed: the presence of exogenous factors (electrolyte disturbances, drugs, hormones and body temperature), different sodium channel kinetics, haplo-insufficiency and co-inherited genetic variants [9]. Recently there has been more interest in these co-inherited genetic variants such as genes encoding regulatory proteins but also genetic variations in SCN5A itself. Genetic variation between individuals is caused by structural variation (eg. Translocations, inversions and splice variants), epigenetic variation (eg. methylation of cytosines) and DNA sequence variation, the latter being the most important factor. One type of DNA sequence variation is the replacement of one nucleotide by another and this is called a single nucleotide polymorphism (SNP). An allele is defined as a genetic locus where more than one genetic sequence has been described. An allele is called a polymorphism if it's frequency exceeds 1% in the general population; it is called a variant if the frequency is lower than 1%. However, the terminology mutation, variant and polymorphism are used on the level of the gene, as well as on the level of the phenotype. A mutation often has a negative connotation: they are true disease causing mutations. In contrast a neutral polymorphism or variant is a genetic sequence variation with no effect on the phenotype (e.g. a silent mutation). Certain polymorphisms or variants cause a change in gene function, but their effect on the phenotype is only visible in combination with other genetic or environmental factors. This last group is called functional polymorphisms. Mutations and neutral polymorphisms can thus be regarded as 2 extremes of a broad spectrum. In this article we describe a family with a mutation in SCN5A and a new variant in SCN5A not described in literature before. Individuals with the combination of the mutation and the variant in this family present with a more severe phenotype including spontaneous atrial tachyarrhythmia during childhood.

## Case (Table 1)

### Index patient (Sibling 3)

**Figure 1:**
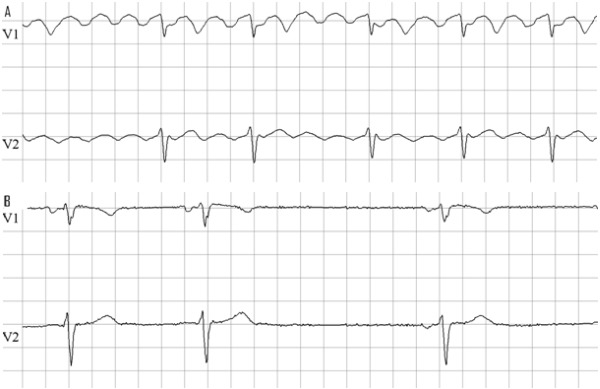
ECG of sibling 3; A: at admission atrial flutter with variable conduction 4/1-3/1; B: after cardioversion showing sinus bradycardia and arrhythmia. (25mm/s; 10mm/mV)

A 14-year old girl (sibling 3) presented to our institution with fast palpitations during exercise lasting a couple of minutes and resulting in pre-syncope, heavy transpiration and retrosternal pain. Her medical history revealed a short loss of consciousness at age 7, which was not further investigated. Electrocardiogram (ECG) showed type 1 atrial flutter with variable conduction (3-4/1) (Figure 1A). Successful electrical cardioversion was performed. Holter monitoring after cardioversion revealed bradycardia (as low as 30 beats per minute (BPM)) and multiple sinus pauses up to 4.4 seconds. There was also an obvious sinus node arrhythmia (Figure 1B). Underlying structural defects were ruled out by transthoracic echocardiogram (TTE) and magnetic resonance imaging (MRI) of the heart. Screening for an infectious (including Borrelia) or auto-immune cause was negative. Family history includes a maternal uncle with pacemaker implantation at age 27 because of recurrent syncopes since age 12 due to SSS and ultimately 3th degree atrio-ventricular (AV) block suggesting progressive cardiac conduction slowing. A dual chamber pacemaker was implanted in the index patient for possible familial SSS (tachy-brady).

Nine months later she presented to the pacemaker consultation because of recurrent exercise related presyncope. Interrogation of the pacemaker and ECG revealed relapse of atrial flutter. She successfully underwent flutter ablation at the cavo-tricuspidal isthmus. Electrophysiological study (EPS) revealed a slight prolongation of the his-ventricle conduction interval. No other conduction abnormalities were noted, nor could ventricular arrhythmias be provoked.

Because of the familial history a genetic examination was performed. She appeared to be carrier of both a mutation R1632H and a variant M858L in the SCN5A gene.

### Probands

**Figure 2:**
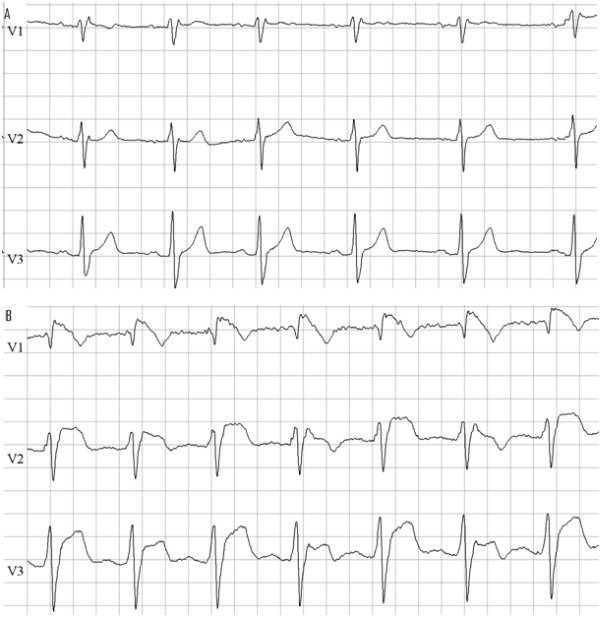
ECG of mother before (A) and after (B) ajmaline infusion with induction of typical Brugada type 1 pattern. (25mm/s; 10mm/mV)

Her mother was identified to be the source of the mutation R1632H in SCN5A. She was known for multiple syncopes highly suggestive for vasovagal syncope. However, infusion with ajmaline unmasked a type 1 Brugada ECG (Figure 2). An EPS was performed: ventricular arrhythmias could not be induced. She was implanted with an implantable loop recorder (ILR). Symptomatic sinuspauses and sinsusbradycardia were detected and a dual chamber pacemaker was implanted. Because of the absence of a spontaneous type 1 ECG, the negative EPS and the fact no ventricular arrhythmia was documented by the ILR we felt there was no indication for an ICD.

Her father was the carrier of the variant M858L. Familial history for sudden cardiac death or arrhythmias was negative. He had a personal medical history of a single syncope in unclear circumstances. Ajmaline provocation was negative. Because of the syncope an EPS was performed. The EPS was negative and a conservative strategy was proposed. Because of one recurrence of syncope rather suggestive for vasovagal syncope an event recorder was implanted. Until now no arrhythmia was documented.

**Figure 3:**
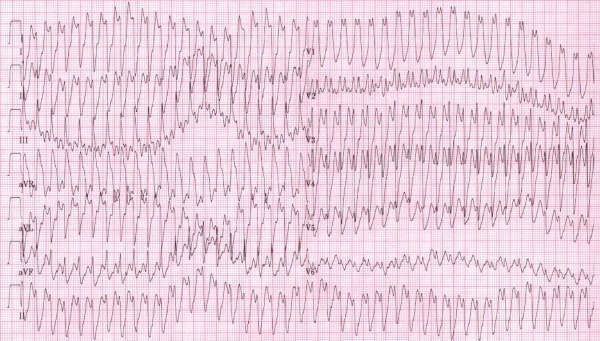
ECG of sibling 4 during exercise test on a treadmill provoking transition to broad QRS tachycardia due to 1/1 conduction of atrial flutter with aberration. There is diffuse intraventricular conduction slowing due to the SCN5A mutation resulting in a tracing resembling ventricular fibrillation. (25mm/s; 10mm/mV)

Her younger brother (sibling 4) was seen in the emergency room a few months after her first presentation with an atrial flutter (3/1 conduction) resulting in a ventricular rhythm of 105 BPM. An exercise test on a treadmill provoked broad QRS tachycardia at 250 BPM after 3 minutes of exercise because of 1/1 conduction of the atrial flutter with aberration (Figure 3). He underwent flutter ablation. After the procedure there was absence of sinus rhythm, atrial standstill and a slow nodal escape rhythm (35 BPM). The diagnosis of familial sick sinus syndrome was withheld. A ventricular stimulation protocol was negative. A DDD-pacemaker was implanted. Genetic testing revealed identical genotype abnormalities as found in his sister: he is also carrier of the R1632H mutation and of the variant M858L in the SCN5A gene.

Her oldest sister (sibling 1) had a history of recurrent fainting with prodromes and palpitations. Prolonged Holter monitoring showed sinus arrhythmia. After a negative ajmaline provocation test and EPS, an ILR was implanted because of the alarming personal and familial history. The ILR revealed third degree atrio-ventricular (AV) block and sinus pauses of more than 3 seconds at time of syncope. The ILR was explanted and a dual chamber pacemaker was implanted. She is carrier of the mutation R1632H in SCN5A.

Her other brother (sibling 2) is asymptomatic up until now. He is carrier of the mutation R1632H in SCN5A. Ajmaline provocation and EPS were negative; a conservative strategy was taken and meticulous follow-up was proposed.

## Discussion

We hypothesize that in this family the combination of both the variant and the mutation in one individual (compound heterozygosity) produced a more severe phenotype of SSS, with spontaneous atrial tachyarrhythmias in childhood. We review cardiac sodium channel function, the different phenotypes caused by SCN5A mutations and focus on genetic variants as a possible explanation for the phenomena of reduced penetrance and variable expression.

### Cardiac sodium channel function and structure with regard to R1632H and M858L

Voltage-gated sodium channels are dynamic transmembrane proteins that open and close to conduct sodium ions. They contain a pore-forming ion-conducting α-subunit and ancillary β-subunits and several regulatory proteins [10]. As already stated, the α-subunit protein is called Nav1.5 and is encoded by the SCN5A gene. It consists of four domains, each domain consists of 6 transmembrane segments. The channel is responsible for the fast upstroke (phase 0) of the cardiac action potential. Time-dependent transitions between distinct conformational states of the channel protein is called gating and is due to molecular movements in response to membrane potential changes (voltage dependent gating) [11]. We can distinguish between activation and inactivation (fast, intermediate and slow). Inactivation starts simultaneously with activation, but since inactivation is a slower process there is a transient conduction of sodium ions during phase 0 of the action potential. Simply put, mutations can cause biophysical gain of function, loss of function or both. Gain of function (as seen in LQTS-3) gives rise to prolonged action potential duration and the possibility of early afterdepolarizations that can induce torsades de pointes [12]. The mechanism underlying gain of function is a sustained sodium current (I-sus) during the plateau phase of the action potential caused by fast reopening due to delayed inactivation causing increased late sodium current [9]. Loss of function (as seen in BrS, PFHB, and SSS) causes a slower upstroke of phase 0 of the action potential. It might occur because of defective trafficking of the mutant α-subunit, slower recovery from inactivation, hyperpolarizing shift of inactivation, depolarizing shift of activation all generating reduced upstroke velocity of the action potential due to reduced current density [9,11,13].

Alternative splicing of SCN5A creates different isoforms of the α subunit. Isoform Nav1.5 is the predominant α subunit in the human heart, but four other functional isoforms have been described so far (Nav1.5a,c,d and e respectively) [14]. Functional consequences vary from unchanged electrophysiological properties to altered kinetics or even nonfunctional channels. It was demonstrated that the T1620K mutation, a mutation known to cause both LQT3 and PFHB,can create different functional effects in the background of different splice variants and that these splice variants might thus play a role in the genotype-phenotype relationship [15].

Nav1.5c is the most commonsplice variant in the human heart and lacks a glutamine at position 1077 [16]. Common human polymorphisms have different electrophysiological properties in the background of Nav1.5c compared to Nav1.5 [17]. Nav1.5e is a neonatal isoform which is downregulated after birth which indicates different expression depending on age [14].

Amino acid 1632 of Nav1.5 is located in the fourth segment of domain 4. The fourth segment of each domain is positively charged and forms the voltage sensor. This voltage sensor is a highly conserved region. It is not located in a possible alternatively spliced region.The missense mutation R1632H has been associated with SSS and PFHB [4]. Two similar electrophysiological studies on R1632H have been performed with similar results [4,18]. There are 2 major abnormalities. First there is a negative shift of steady state inactivation towards hyperpolarized potentials resulting in reduced channel availability. Secondly, recovery from inactivation was dramatically decelerated resulting in an inactivated channel at physiological membrane potentials and normal heart rates. Our report is the first to link R1632H with BrS [19,20]. R1632H has thus so far been linked to SSS, PFHB and BrS. Whether it is possible to identify loss of function SCN5A mutations that exclusively result in one phenotype, despite sharing similar biophysical properties, remains a matter of debate.

Amino acid 858 of Nav1.5 is located in the fifth segment of domain 2. It is not located in a possible alternatively spliced region. Segment 5 and 6 of each domain form the ion-conducting pore of the channel. Mutations in this region have been associated with different phenotypes of SCN5A disease. M858L has not yet been described in the literature before. Our hypothesis is that this variant is a modifier of the SCN5A-related channelopathy described in this family.

### Phenotypical expression of cardiac sodium channel disease

As already stated, mutations in SCN5A are responsible for several distinct clinical entities. The first clinical entity associated with a mutation in SCN5A was LQTS in 1995 [2]. Since then, mutations in all domains of SCN5A have been associated with LQTS, BrS [1], PFHB [3], SSS [4], DCM [5] and l-AF [6]. Overlap syndromes have also been described [7]. It has been suggested not to view SCN5A mutation carrying patients as having multiple separate disease entities, but as suffering from one disease designated 'SCN5A syndrome' [21].

#### Brugada syndrome

The Brugada syndrome was first described and designated as such in 1992 by the brothers Pedro and Josep Brugada [22]. Although a similar disease entity was already described in 1989 by Martini et al. [23]. Originally the Brugada syndrome was diagnosed in subjects with high risk for sudden cardiac death due to ventricular tachyarrhythmias (polymorphic ventricular tachycardia (pVT) and ventricular fibrillation (VF)), accompanied by typical coved-type ST-segment elevation in the right precordial leads V1 to V3. Loss of function mutations in SCN5A have been linked with BrS [1]. The incidence of BrS is around 1 in 8000. Approximately 20% of BrS patients have a mutation in SCN5A and almost 300 different mutations in SCN5A have been described [19,20]. Mutations in regulatory proteins and beta subunits of the cardiac sodium channel and mutations in other cardiac ion channels, such as calcium and potassium channels have also been reported [19].

In the report of the second consensus conference on Brugada syndrome the diagnostic criteria and therapeutic considerations for BrS were updated [24]. According to these new criteria Brugada syndrome is definitively diagnosed when a type 1 ST-segment elevation (≥2 mm, negative T) is observed in >1 right precordial lead (V1 to V3) in the presence or absence of a sodium channel blocking agent, and in conjunction with one of the following: documented VF, polymorphic VT, a family history of sudden cardiac death at < 5 years old, coved-type ECGs in family members, inducibility of VT with programmed electrical stimulation or syncope. It is interesting to notice neurally mediated syncope is more common in patients with BrS, probably due to an impaired balance of the autonomic nervous system [25]. The ECG manifestations may be concealed at a particular moment in time. They can be unmasked by fever [26], vagotonic agents [27], superior placement of the ECG electrodes [28] and administration of a sodium channel blocker [29]. Drug challenge with sodium channel blockers such as ajmaline and flecainide is used in practice to unmask a type 1 ECG.

Symptomatic (syncope, aborted sudden cardiac death, documented VT/VF) BrS patients should get an implantable cardioverter defibrillator (ICD) [24]. Choosing the right therapy is much more difficult in asymptomatic BrS. The report of the second consensus conference on Brugada syndrome attributed a great value in risk stratification to whether or not VT could be induced in EPS [24]. However, two recent multicenter studies have questioned the importance of inducibility of VT in EPS [30,31]. Because of these findings and the rate of complications (mainly inadequate shocks) in patients with an ICD, there is increasing reluctancy to injudicious ICD implantation in BrS. We summarize clinical and ECG parameters associated with a worse outcome in Table 2. Signal averaged ECG measurement to determine fragmented QRS [32] and late potentials [33,34] is a widely available non-invasive test that has proven its usefulness in risk stratification. Genetic data might be proposed in the future as a new tool for risk stratification, as mutations causing premature truncation of the protein or causing inactive proteins seem to produce a more severe phenotype [35]. There are strong clinical and experimental data suggesting quinidine could be a good alternative therapy for asymptomatic BrS, nevertheless more studies are needed [36].

#### Progressive familial heart block

According to the 'Online Inheritance In Men' (OMIM) library, PFHB is the correct term for the disease formerly known as progressive cardiac conduction defect, which is also called Lev-Lenegre disease. Therefore we encourage this entity to be called PFHB. It is the prevailing phenotype in carriers with loss of function mutations in SCN5A [376]. This defect is characterized by progressive alteration of impulse propagation through the His-Purkinje system, with right or left bundle branch block and widening of the QRS complex, resulting in complete atrio-ventricular block, syncope and sometimes even sudden death. In vitro studies have demonstrated that properties of loss of function include reduced density of the sodium current and altering of the gating properties [38,39]. Information obtained from a mouse model shows age-related channelopathy-mediated fibrosis of the myocardium [40]. These findings support the hypothesis suspecting a fibrotic process to be responsible for slowly progressive AV block. The first association of SCN5A mutations with PFHB originated from the description of a novel mutation causing either PFHB or BrS in the same family [3,41]. Because of the age-related progression and the possible severity of the defect, it is advised that carriers of a SCN5A mutation get a yearly clinical and ECG follow-up [37]. Pacemaker implantation is indicated for advanced second degree and third degree AV block [42].

#### Sick sinus syndrome

SSS comprises different forms of arrhythmia that result from sino-atrial node and atrial dysfunction. It is diagnosed by ECG and characterized by variable manifestations including sinus bradycardia, sinus arrest, atrial standstill, and tachy-brady syndrome (supraventricular tachycardia alternating with episodes of sinus bradycardia) [43]. In the first description of SCN5A related SSS, the authors suggested an autosomal recessive pattern of inheritance because SSS was associated with compound heterozygous loss of function mutations, for example R1632H + delF1617 [4]. However this hypothesis was contested a couple years later when a novel mutation was found that resulted in an autosomal dominant pattern of inheritance of SSS [44]. Mutations in SCN5A have been demonstrated to impair the coupling of electrical events between the pacemaker cells and the cells surrounding them within the sino-atrial node, and cause impairment of propagation of electrical activity from the sino-atrial node to atrial tissue [45]. Pacemaker implantation is the treatment of choice for symptomatic sinus node dysfunction [42]. Tachyarrhythmias should be treated according to the most recent guidelines [46].

#### Dilated cardiomyopathy

Dilated cardiomyopathy (DCM) is a primary myocardial disease characterized by dilatation of the left or both ventricles and impaired systolic function, which may proceed to congestive heart failure [47]. Up to 50% of dilated cardiomyopathies are idiopathic and 20 % of these display familial prevalence [48]. Mutations in genes encoding various proteins that are involved in the contractile apparatus and cytoskeleton were found in these families [48]. It was only in 2004 that a mutation in SCN5A was linked with DCM, thereby expanding the spectrum of SCN5A associated diseases [5]. Most of these mutations are found in patients who display multiple phenotypes. The biophysical effects of these mutations are often both a combination of gain and loss of function [49]. The pathophysiology of DCM caused by SCN5A mutations still remains unclear. There are however two hypotheses. Ge et al speculated that the interactions between cardiac sodium channels and intracellular proteins are disrupted, thereby disturbing normal cardiomyocyte structure and function [49]. On the other hand it is known that gain of function mutations lead to a higher influx of sodium and an increased intracellular sodium concentration with a secondary increase in intracellular calcium. This is associated with cellular remodeling and development of hypertrophy and heart failure [50]. Therapy for SCN5A associated DCM exists of standard therapy for congestive heart failure.

#### Long QT syndrome type 3

Long QT syndrome is an inherited arrhythmogenic disease characterized by prolongation of the QT interval, the ECG equivalent of an increased action potential duration in ventricular cardiomyocytes and thus delayed ventricular repolarization. The limit for corrected QT interval as calculated by the formula of Bazett is 470 milliseconds for females and 450 milliseconds for males [51]. Prolongation of the QT interval due to increased late sodium current predisposes to early afterdepolarizations that induce torsades de pointes, which may result in recurrent syncope or sudden cardiac death [9,12]. Among the numerous different genetic subtypes (all encoding a protein that is directly or indirectly involved in repolarization) of the syndrome, LQT-3 is caused by gain-of-function mutations in SCN5A. Inherited LQTS has an estimated incidence of 1 in 3000 and LQT-3 has a relative prevalence of 10% [52]. So far more than 80 different SCN5A mutations have been described [53]. In contrast to the other LQTS subtypes, patients with LQT-3 tend to experience cardiac events during sleep and rest when the heart rate is slow, rather than during exercise or at times of emotions seen in other types of LQTS [54]. Standard treatment for LQT-3 include potassium substitution, spironolactone and ICD implantation. Recently however, 2 drugs that target the pathophysiology of LQT-3 have come available. Mexiletine attenuates the increased inward sodium current, thereby abbreviating the QT interval [55]. Ruan et al. showed mexiletine has mutation specific effects [56]. It can exacerbate QT prolongation in some mutations so caution should be used when recommending mexiletine to carriers of mutations with undefined electrophysiological properties [57]. Ranolazine is a selective blocker of late sodium current and abbreviates the QTc interval in a dose dependent manner [58,59]. Although its promising properties, larger clinical trials are needed to confirm the efficacy of ranolazine.

#### Atrial Fibrillation

Atrial fibrillation in the absence of risk factors (advanced age, diabetes mellitus, arterial hypertension, structural heart disease and congestive heart failure) is a condition known as lone atrial fibrillation (l-AF) [60]. Lone AF has been associated with mutations in genes encoding potassium channels and more recently with mutations in SCN5A [6,61]. Both gain and loss of function mutations in SCN5A are linked to l-AF. BrS has been associated with an increased risk for AF. Amin et al showed that loss of function SCN5A mutations in BrS are associated with intra-atrial conduction slowing, a substrate for AF maintenance, and decreased atrial ectopic activity, which may inhibit the trigger for AF initiation [62]. Similar to what is observed in LQT-3, gain of function mutations result in increased late sodium current and prolongation of the action potential duration thereby triggering early afterdepolarizations, not only in ventricular cardiomyocytes but also in atrial cardiomyocytes. Flecainide is the drug of choice in AF caused by gain of function SCN5A mutations and in prevention of atrial arrhythmias in LQT-3 [63,64].

#### Early repolarization syndrome

Early repolarization is characterized by an elevation of the junction between the end of the QRS complex and the beginning of the ST segment (J point) from baseline on ECG. For decades it has been considered to be a benign ECG manifestation. However, recently it has been associated with idiopathic VF and sudden cardiac arrest in a large clinical trial [65]. Up until now, one abstract has been published linking SCN5A mutations with early repolarization syndrome [66]. The exact role of SCN5A in ERS has to be further investigated.

#### Overlap syndromes

Overlap syndromes have been described; they involve clinical symptoms or arrhythmias of the various presentations in one patient (BrS, PFHB, LQTS, SSS, l-AF and DCM). This description is also used when a single mutation causes various arrhythmogenic phenotypes in different families or members of one family. Because of this overlap, Remme et al. propose to call patients who possess an SCN5A mutations as having 'SCN5A syndrome or disease' with the different phenotypical expressions as a subtype of this disease [21].

It is plausible to understand overlap exists between phenotypes caused by mutations with similar functional abnormalities (loss of function mutations and gain of function mutations respectively). Multiple reports have been published [41,63,67-69]. However, overlap between LQTS and phenotypes associated with loss of function mutations have also been described [7,70,71]. Studies on heterologous expression systems have shown that one mutation can exhibit both loss of function and gain of function properties, thereby providing an explanation for these at first sight rather strange overlap syndromes [7,71]. Another possible explanation is the presence of a specific variant in the background of an alternatively spliced Nav 1.5 protein generating functionally distinct channels [15]. Choice of treatment is dependent on the prevailing clinical phenotype.

### Variable expression, reduced penetrance and the importance of genetic variants

Co-inherited genetic variants are a major point of interest for researchers to explain the phenomenena of variable expression and reduced penetrance. Genetic modification by genetic variants may occur in the disease gene locus itself or in a genetic locus apart from the disease gene locus, for example in genes encoding ancillary β-subunits or genes encoding one of the several regulatory proteins [15].

The spectrum and prevalence of non-synonymous (inducing a change in amino acid or stop codon) genetic variants in the cardiac sodium channel was determined among 829 healthy subjects from different ethnicities [72]. Another research group did the same in a Japanese population consisting of 166 arrhythmic patients and 232 healthy controls [73]. Variant M858L was not found in these studies, and has not been described in literature before.

Research on genetic variants is done through clinical studies preferably in large families, electrophysiological studies (eg. patch-clamp studies on heterologous expression systems) and transgenic animal models. Induced pluripotent stem cells (iPS) are a promising new technique. Research remains necessary to unravel the role of genetic variants in channel function and their correlation with disease.

SNP H558R is the most prevalent and most investigated genetic variant in SCN5A but was not found in either of the family members in the case described. Studies have shown that the presence of the less common allele G attenuated the ECG characteristics of the Brugada syndrome among carriers of an SCN5A mutation [74]. There is also evidence for mutation-specific effects on SCN5A related SSS (the R558 variant caused aggravation of the defect in some mutations and correction of the defect in others) [75], stabilization of channel fast inactivation in a certain gain-of-function mutation [76] and rescuing defective trafficking of the Nav1.5 protein caused by a loss of function mutation [77]. Other variations in the genome and their effects on SCN5A mutations that have been investigated include SNP R1193Q [17,78,79], splice variant Q1077 [17] and SNP S1103Y [17,80]. All the observations done in these studies provide a plausible mechanism for decreased or increased arrhythmogenic events in patients who not only carry mutants but also carry specific cardiac sodium channel variants that act as functional polymorphisms. We presume a similar interaction between mutation R1632H and variant M858L in the family we described causing increased arrhythmogenic events (atrial tachy arrhythmias).

Although co-inherited genetic variants are extremely interesting and important in understanding reduced penetrance and variable expression, we must also be cautious because not every genetic variant plays a role in disease expression. We currently do not have guidelines for determining from what point on certain anomalies in electrophysiological studies are clinically important. The reliance on statistically significant functional differences, certainly when they are subtle, as the sole criterion for claiming that a sequence variant is a disease causing mutation has been put into question [81]. Sorting signal from noise as postulated by Milan et al is an intriguing challenge [81].

Another interesting issue regarding SCN5A mutations is reduced penetrance. Oliva et al. therefore suggested that it should be avoided seeing a mutation as a certain predictor of sudden death, but rather as a risk factor [82]. Probst et al. take it even a step further: in families with a known SCN5A mutation they found 8 individuals with phenotypical familial BrS without carrying the familial SCN5A mutation [83]. Therefore they suggested that modulating factors within the studied families (genetic background) are sufficiently powerful to evoke a BrS positive ECG. This genetic background includes all proteins and molecules that play a role in the pathophysiology of BrS, or by extension the pathophysiology of all possible phenotypical expressions of SCN5A mutations.

### Limitations of the study

We describe a single family where the combination of a known mutation and a new variant seems to produce a more severe phenotype than the mutation per se. This aggravation was not due to age related factors since the youngest siblings were those suffering from atrial tachyarrhythmia (Table 1). Of course, reduced penetrance and variable expression of R1632H in this family can possibly explain the gravity of the phenotype seen in the two children with atrial tachyarrhythmia. Furthermore since we did not perform cellular electrophysiological studies on R1632H or M858L or the combination, we can't prove our hypothesis with absolute certainty. More clinical or cellular data concerning M858L are needed to definitely consider this variant as a functional or pathogenic polymorphism.

## Conclusion

SCN5A mutations are prone to variable expression and reduced penetrance. The different phenotypes include not only BrS and LQTS, but also PFHB, SSS, l-AF and DCM, each of them with a specific risk for sudden cardiac death, treatment and follow-up. Genetic variation in the disease-causing gene or in other genes involved in the pathophysiology of the different phenotypes is one of the most important explanations for the phenomena of reduced penetrance and variable expression.

We described a family where some individuals possess a combination of a loss of function mutation and a variant in SCN5A. In the literature the mutation R1632H was not yet associated with the BrS phenotype. The variant M858L has not been described in literature before. The individuals carrying the mutation and the variant had a more severe phenotype including atrial tachyarrhythmia in chidhood in comparison with individuals carrying only the mutation. Since this variant has not been described in large population studies of genetic variants in the SCN5A gene and the specific phenotypic findings, it seems reasonable to assume that the M858L variant might be a modifier in the SCN5A-related channelopathy described in this family. However further studies are needed to investigate whether M858L is a true pathogenic mutation, a functional polymorphism or just a neutral polymorphism.

## Figures and Tables

**Table 1:**
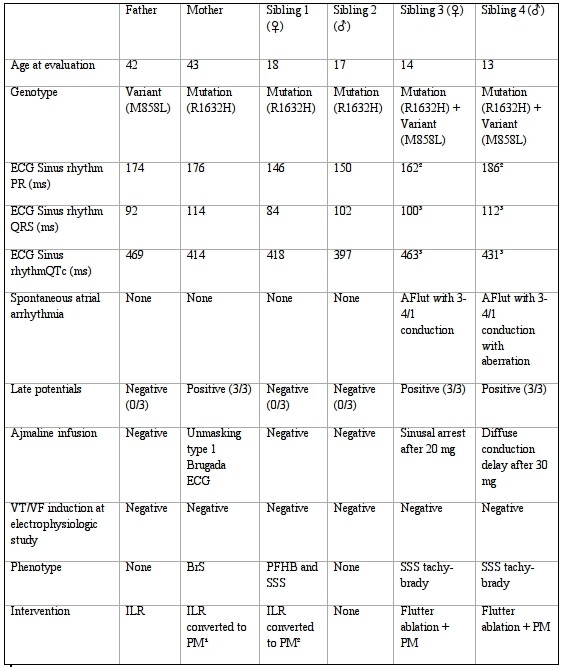
Summary of the index patient and family

1= symptomatic sinus pauses and sinus bradycardia; 2= syncope with documentation of third degree AV block and sinus pauses; 3= ECG after cardioversion; VT: ventricular tachycardia; VF: Ventricular fibrillation; BrS: Brugada syndrome; ILR: implantable loop recorder; PFHB: progressive familial heart block; SSS: sick sinus syndrome; PM: pacemaker; AFlut: atrial flutter

**Table 2:**
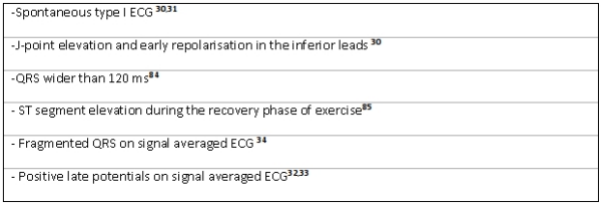
Predictors of worse outcome in asymptomatic BrS
